# The assessment of tumor-infiltrating lymphocytes in invasive apocrine carcinoma of the breast in relation to the HER2 status

**DOI:** 10.17305/bb.2023.9868

**Published:** 2024-04-01

**Authors:** Zoran Gatalica, Nataliya Kuzmova, Inga Rose, Monika Ulamec, Melita Peric-Balja, Faruk Skenderi, Semir Vranic

**Affiliations:** 1 Reference Medicine, Phoenix, Arizona, USA; 2 The University of Oklahoma Health Sciences Center, Oklahoma, USA; 3 Ljudevit Jurak Clinical Department of Pathology and Cytology, Sestre Milosrdnice University Hospital Center, Zagreb, Croatia; 4 Department of Pathology and Scientific Group for Research on Epigenetic Biomarkers, University of Zagreb School of Medicine, Zagreb, Croatia; 5 Oncological Pathology Department, Ljudevit Jurak Clinical Department of Pathology and Cytology, Sestre Milosrdnice University Hospital Center, Zagreb, Croatia; 6 Department of Pathology, Sarajevo School of Science and Technology, Sarajevo, Bosnia and Herzegovina; 7 College of Medicine, QU Health, Qatar University, Doha, Qatar

**Keywords:** Breast cancer, special types, apocrine carcinoma, tumor-infiltrating lymphocytes (TILs), HER2-low

## Abstract

In the current study, we assessed the prevalence and molecular features of HER2-low phenotype in the apocrine carcinomas of the breast (ApoCa) and its relationship with tumor-infiltrating lymphocytes (TILs). A cohort of 64 well-characterized therapy-naïve ApoCa was used. The TIL distribution was assessed using the hematoxylin and eosin whole slide/scanned images following the international TILs working group recommendations. Next-generation sequencing (NGS) was performed in a subset of HER2-low ApoCa. All patients were women, with a mean age of 62 years. Forty-three carcinomas were pure apocrine carcinoma (PAC; ER**−**/AR**+**), and the remaining 21 were classified as apocrine-like carcinomas (ALCs; ER**+**/**−**, AR**+**/**−**). HER2/neu was positive (score 3**+** by IHC and/or amplified by FISH) in 20/43 (47%) PAC and 4/21 (19%) ALC. The prevalence of HER2-low expression (scores 1**+** or 2**+** without *HER2* amplification) in ApoCa was 39% without significant differences between PAC and ALC (*P*** ═ **0.14); however, the HER2-low phenotype was more prevalent in triple-negative PAC than in ALC (*P* < 0.001). Levels of TILs were low (≤10%) in 74% of ApoCa (median: 5%, range 0%–50%). TIL levels were significantly higher in ALC than in PAC (*P* ═ 0.02). HER2 status had no impact on TIL distribution (*P* ═ 0.45). The genomic profile of HER2-low ApoCa was similar to other subtypes of ApoCa. ApoCa has predominantly low TIL, particularly PAC. The prevalence of the HER2-low phenotype in ApoCa is high, which should have therapeutic and clinical implications given the recently approved therapies with antibody–drug conjugates (ADCs) for HER2-low breast cancers.

## Introduction

Apocrine carcinoma of the breast (ApoCa) is a rare (∼1%), special type of breast cancer with characteristic apocrine morphology and steroid receptor expression profile: estrogen receptor (ER)-negative and androgen receptor (AR)-positive [1, 2]. Most ApoCa are categorized as triple-negative carcinomas; however, *HER2/neu* overexpression (score 3+) has been reported in 30%–50% of ApoCa caused by the *HER2/neu* gene amplification [1, 3–6]. Recently, HER2-low breast cancers, defined by HER2 scores 1+ or 2+ without *HER2/neu* gene amplification, have come into clinical focus due to the marked therapeutic efficiency of trastuzumab deruxtecan (DS-8201) in metastatic, HER2-low breast cancers [7]. Trastuzumab deruxtecan (DS-8201) is an antibody–drug conjugate (ADC) composed of a humanized anti-HER2 monoclonal antibody linked to the topoisomerase I inhibitor payload via a linker [8, 9]. The prevalence of HER2-low expression in metastatic breast cancers of no special type (NST) is ∼60% but has not yet been specifically explored in ApoCa [10].

Tumor-infiltrating lymphocytes (TILs) play a pivotal role in mediating response to cytotoxic chemotherapy and improving the clinical outcomes of breast cancer patients [11]. TIL-enriched breast cancers, mainly ER-negative carcinomas, may also be more responsive to immunotherapy with immune checkpoint inhibitors (ICIs) [12]. The evaluation of stromal TILs has been a subject of controversy due to their subjective morphologic assessment. However, substantial efforts have been undertaken to improve and standardize the TIL assessment in breast cancer. Thus, the International TILs Working Group 2014 published recommendations and practical guidelines to enable more reliable and reproducible assessment of stromal TILs in breast cancer [13]. Numerous studies have explored and confirmed the clinical relevance of stromal TILs in breast cancer, leading to incorporating this parameter into the routine assessment of all early triple-negative breast cancers (TNBCs) as a prognostic biomarker (Level 1B evidence) [14]. However, the St. Gallen International Breast Cancer Consensus 2021 and the current breast cancer clinical guidelines do not endorse a routine TIL assessment in breast cancer, nor do they recommend the de-escalation of chemotherapy based on the TIL status [15]. On the other hand, the status of stromal TILs in ApoCa has been rarely analyzed with limited available data (reviewed in [16]).

In the current study, we investigated the status of TILs in a cohort of ApoCa, and also analyzed the prevalence and molecular features of the HER2-low phenotype in ApoCa and its impact on TILs.

## Materials and methods

### Sample collection

Formalin-fixed paraffin-embedded (FFPE) surgical samples and/or microscopic slides of ApoCa cases were retrieved from the Department of Pathology at The University of Oklahoma Health Sciences Center (*n* ═ 6) [17], the Ljudevit Jurak Clinical Department of Pathology and Cytology at Sestre Milosrdnice University Hospital Center (Zagreb, Croatia) (*n* ═ 7) [18], and from the authors’ consultation cases (*n* ═ 51) collected during their diagnostic breast pathology services. All cases were graded using the Nottingham histologic grade and re-reviewed for the current study [19]. The status of ER, progesterone (PR), and HER2/neu receptors was routinely utilized during the diagnostic work-up, following the international CAP/ASCO guidelines [20, 21]. HER2-low was defined as scores 1+ and 2+ by IHC without *HER2/neu* gene amplification, as determined by in situ hybridization assays [21]. In addition, immunohistochemical androgen receptor (AR) expression was utilized for the molecular classification of ApoCa [3, 4, 14]. The tumors were considered positive for AR if they exhibited positivity in ≥10% of the cancer cells’ nuclei [3].

### Molecular classification of apocrine carcinomas (ApoCa)

As earlier proposed, all ApoCa cases were classified based on their characteristic morphology and steroid receptors status: cases that were ER-negative and AR-positive were classified as pure apocrine carcinomas (PACs), while the remaining cases with variable expression of ER and AR (ER+/−, AR+/−) were classified as apocrine-like carcinomas (ALCs) [3, 4, 17].

### Assessment of tumor-infiltrating lymphocytes (TIL)

TILs were assessed in the stromal compartment, denoted as percentage stromal TILs (% stromal TILs). The assessment excluded TILs located outside the tumor borders, around in situ components, and within normal ducts/lobules. The areas affected by the crush artifacts, tumor necrosis, extensive stromal hyalinization, and the previous core biopsy sites were also excluded. Only mononuclear cells (lymphocytes and plasma cells) were scored, while polymorphonuclear leukocytes were excluded. The stromal TILs were reported as a continuous variable [13]. The average stromal TIL percentage was calculated for samples with two or more slides available. In cases of intratumoral heterogeneity, different areas were scored, and the average was taken as the final percentage of stromal TILs [13]. TIL assessment was conducted using either glass slides or scanned images. Slides were scanned using the Philips Intellisite Pathology Solution Ultra Fast Scanner^®^ (Philips Medical Systems Nederland B.V.) and reviewed using Aperio ImageScope^®^ 12.3 version (Leica Microsystems, Wetzlar, Germany).

### Next-generation sequencing for HER2-low ApoCa

Next-generation sequencing (NGS) was performed on selected ApoCa cases using the commercially available platform of Caris Life Sciences (Phoenix, AZ, USA), and the method was previously reported [3, 22–24].

### Ethical statement

All cases were de-identified and pseudo-anonymized for the purposes of this study. For samples sourced from the University of Oklahoma College of Medicine, approval was granted by the Institutional Review Board of the University of Oklahoma (Approval number: IRB#12866), while the Croatian samples received approval from the Ethical Committee of the Sestre Milosrdnice University Hospital Center (Approval number: EP-13659/21-11).

### Statistical analysis

Pearson’s chi-squared test explored statistically significant differences between the expected and the observed frequencies in categorical variables. For 2 × 2 contingency tables, Fisher’s exact test was used. All statistical analyses were performed using the IBM Statistical Package for the Social Sciences (IBM SPSS, version 27). Statistical significance was set at *P* < 0.05.

## Results

### Clinicopathologic characteristics of the ApoCa cohort

The demographic and pathologic characteristics of the ApoCa cohort are summarized in Table 1. All 64 patients were women diagnosed with primary, treatment-naïve ApoCa. The mean age of the patients was 62.4 years, with an age range of 31–83 years. All ApoCa cases were categorized as either grade 2 (44%) or grade 3 (56%) carcinomas. Based on the steroid receptor profile, 43 ApoCa cases (67%) were classified as PAC, while 21 ApoCa cases were identified as ALCs (Table 1).

**Table 1:** Overview of the major demographic and pathologic characteristics of the ApoCa cohort

**Characteristic (Parameter)**	**Results (*n* ═ 64 cases)**
Age (mean, range)	62.4 years (31–83 years)
Sex	Female (64, 100%)
Subtypes	*Pure apocrine carcinomas (ER−/AR+):* 43/64 (67%): 23 triple-negative and 20 HER2/neu positive cases
	*Apocrine-like carcinomas (ER−/+, AR−/+):* 21/64 (33%): 4 HER2/neu positive cases
Tumor grade	*Grade 2:* 28 cases (44%)
	*Grade 3:* 36 cases (56%)
*HER2/neu* status	Negative (15/64, 23%)
	Positive (24/64, 38%)
	HER2-low (25/64, 39%)
TIL status (mean, median, range)	12%, 5%, 0%–50%

TIL: Tumor-infiltrating lymphocytes; ER: Estrogen receptor; AR: Androgen receptor; ApoCa: Apocrine carcinomas of the breast.

### HER2 status in the ApoCa cohort

**Figure 1.**
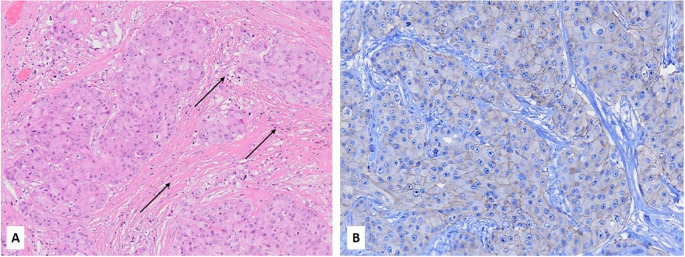
**(A) Hematoxylin and eosin stain of a case of PAC (ER−/AR+) of the breast with low TIL expression (∼1%), as indicated by black arrows (10x); The same case exhibited 2+ HER2 expression by immunohistochemistry (20**x**); (B) A subsequent FISH assay revealed no *HER2/neu* gene amplification (*HER2/CEP17* ratio ═ 1.13).** PAC: Pure apocrine carcinoma; TIL: Tumor-infiltrating lymphocyte; FISH: Fluorescent in situ hybridization.

HER2/neu data were retrieved from the previous histopathologic reports. HER2/neu was positive (score 3+ by IHC and/or amplified by FISH) in 24/64 (37.5%) of ApoCa cases, including 20/43 (47%, 95% CI: 33%–61%) PAC and 4/21 ALC (19%, 95% CI: 8%–40%) (*P* < 0.001). The prevalence of HER2-low expression (scores 1+ and 2+ without *HER2* amplification) in ApoCa was 39% without significant differences between PAC and ALC (37% vs 43%, *P* ═ 0.14); however, the HER2-low phenotype was more prevalent in triple-negative PAC than in ALC (70% vs 43% *P* < 0.001) (Table 2 and Figure 1A and 1B).

**Table 2:** Molecular subtypes of apocrine carcinomas differed significantly concerning HER2 status

		**Molecular subtypes**			**Total**
		**PAC (Triple-negative)**	**PAC (HER2+)**	**ALC**
HER2 status	Negative	7 (30%)	0 (0%)	8 (38%)	15 (23%)
	Positive	0 (0%)	20 (100%)	4 (19%)	24 (37.5%)
	HER2-low	16 (70%)	0 (0%)	9 (43%)	25 (39%)
Total		23 (36%)	20 (31%)	21 (33%)	64

PAC: Pure apocrine carcinomas; ALC: Apocrine-like carcinoma.

### TIL status in the ApoCa cohort

Stromal TILs were fully assessable in 61 out of the 64 cases (95%). The remaining three cases were predominantly apocrine ductal carcinomas in situ (DCIS) with limited invasive component for stromal TIL assessment (microinvasive carcinomas).

**Figure 2.**
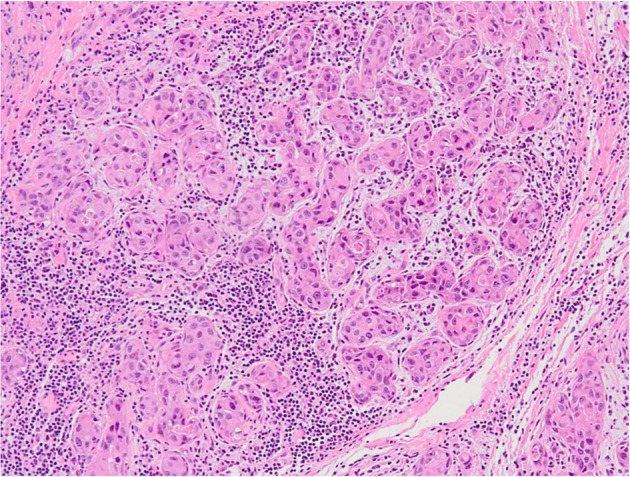
**A case of apocrine-like (ER+/AR+) HER2-low (score 1+) carcinoma with moderate (∼50%) TIL expression (Hematoxylin and eosin, 10x).** TIL: Tumor-infiltrating lymphocyte.

The median of the stromal TILs was 5% (mean 12%), with a range of 0%–50% (Figure 1B). Overall, levels of the stromal TILs in ApoCa were low, with 45/61 (74%) having stromal TILs ≤ 10% (Table 3 and Figure 1A). Only five cases (all ALC) were markedly enriched with stromal TIL (═50%) (Table 3 and Figure 2), and the difference was statistically significant (*P* ═ 0.02). The HER2 status, including HER2-low phenotype, did not impact the stromal TIL distribution (*P* ═ 0.45). Additionally, there was no correlation between the levels of stromal TILs and the patient’s age or tumor grade (*P* > 0.05).

**Table 3:** Levels of tumor-infiltrating lymphocytes according to the molecular subtype of apocrine carcinoma

**Tumor-infiltrating lymphocytes (%)**	**Molecular subtypes of apocrine carcinoma**			**Total**
	**Pure apocrine carcinoma**		**Apocrine-like carcinoma**	
	**Triple-negative**	**HER2+**		
0%	1	1	2	4
1%	1	5	6	12
2%	1	0	0	1
5%	8	4	4	16
10%	7	2	3	12
20%	1	3	1	5
30%	2	4	0	6
50%	0	0	5	5
Total	21	19	21	61

### NGS profile of HER2-low ApoCa

The mutational profile of HER2-low ApoCa was available for five cases of PAC. The most notable pathogenic mutations observed *PTEN* (2/5), *PIK3CA* (2/5), *TP53* (1/5), *BRAF* (1/5), and *KRAS* (1/5) (Table 4).

**Table 4:** Mutational profile of HER2-low ApoCa

**Case**	**HER2 status**	**Molecular subtype**	**Mutational profile**
Case#1	Score 2+ without amplification	PAC	*PIK3CA, H1047R*
Case#2	Score 1+	PAC	*PTEN, P248_V249del, PTEN, Q214X*
Case#3	Score 1+	PAC	*PTEN, P213L, TP53, C141Y*
Case#4	Score 1+	PAC	*PIK3CA, E542K, KRAS, G12D*
Case#5	Score 2+ without amplification	PAC	*BRAF, D594F*

PAC: Pure apocrine carcinomas; ApoCa: Apocrine carcinomas of the breast.

## Discussion

ApoCa is a rare breast cancer subtype with limited targeted therapy options, beyond the anti-HER2 treatment modalities for HER2-positive (3+) cases [14, 16]. Recent breakthroughs in treating advanced, HER2-low (2+) breast cancers with the ADC trastuzumab deruxtecan have been reported [7, 25]. The clinical trial DESTINY-Breast04 (ClinicalTrials.gov number, NCT03734029) revealed significantly longer overall and progression-free survival in the patients with HER2-low breast cancers treated with trastuzumab deruxtecan, compared to those treated with conventional chemotherapy [7]. This led to the Food and Drug Administration (FDA) approval of the drug for all previously treated advanced HER2-low breast cancers [26]. Therefore, recognizing HER2-low breast cancers, particularly in the metastatic setting, has become clinically relevant for the proper management of breast cancer patients. Our study revealed a relatively high prevalence (39%) of HER2-low phenotype in the ApoCa cohort. This prevalence aligns with a recently published systematic review with meta-analysis that revealed an overall HER2-low phenotype prevalence of 36% among >4000 breast cancer patients with TNBC [27]. Therefore, our findings may provide additional, valuable treatment options for a substantial proportion of the patients with advanced ApoCa.

Although limited to only five cases, our mutational profiling revealed a preponderance of ApoCa for mutations within the PIK3CA/PTEN and MAPK signaling pathways [3, 5, 28]. These data indicate that HER2-low ApoCa shares similar molecular features to other ApoCa subtypes and aligns with the recent data from NST carcinomas that revealed that HER2-low is not a distinct clinical and molecular subtype of breast cancer [29–32].

The treatment with ICIs has markedly improved the outcomes of numerous cancer subtypes, including TNBC, with agents like pembrolizumab. Predictive biomarkers for ICI include PD-L1 expression, tumor mutational burden (TMB), and microsatellite instability-high (MSI-H) status. So far, PD-L1 expression on immune cells has been validated as a predictive biomarker in breast cancer. In contrast to the recent study of Ni et al. [33], previous data indicate that ApoCa exhibits low PD-L1 expression, low TMB, and consistently displays MSI stability (reviewed in [5, 16, 28], and [34–36]). High stromal TIL presence has been a feature of ER-negative breast carcinomas, particularly TNBC [37, 38]. Although not entirely validated and approved, high stromal TIL levels may indicate good responses to conventional chemotherapy and ICIs. Additionally, stromal TIL is also a potent prognostic factor associated with favorable clinical outcomes in early-stage TNBC [38]. Specifically, high stromal TIL levels in stage II TNBC is associated with with better outcomes compared to stage I TNBC with lower TIL levels [39]. Three previous studies revealed low stromal TILs in triple-negative ApoCa and a low enrichment with CD3+ and CD8+ lymphocytes, consistent with our results, particularly in PAC [35, 36, 40]. Based on both current data and previous studies, ApoCa patients appear to be less likely responsive to ICIs.

A limitation of our study is the lack of clinical and follow-up information; hence, we could not correlate the status of TIL with the patient’s outcome. We also used the initially proposed recommendations for TIL assessment by the International TILs in Breast Cancer Working Group (now renamed as the International Immuno-Oncology Biomarker Working Group). These guidelines were later revised to include the assessment of the invasive front of cancers, defined as a 1-mm rim around the tumor. The HER2/neu results were derived from previous histopathologic reports and were not subject to central re-review.

## Conclusion

ApoCa of the breast, particularly PAC, does not appear to be enriched with the stromal TIL, which, along with low-PD-L1, low TMB, and microsatellite stable status known from previous studies, make these cancers less amenable to the treatment with ICI. HER2-low APoCa exhibits similar TIL features and mutational profiles to other ApoCa subtypes. However, HER2-low expression in approximately 40% of ApoCa cases provides a valuable predictive biomarker to trastuzumab deruxtecan, opening a new avenue for treating advanced diseases. Further clinical studies (trials) should confirm the clinical relevance of our observations.
